# What helps distressed Australian adolescents impacted by cancer? Mechanisms of improvement of the PEER program

**DOI:** 10.1111/hsc.13873

**Published:** 2022-06-21

**Authors:** Pandora Patterson, Fiona E. J. McDonald, Helen Bibby, Kimberley R. Allison

**Affiliations:** ^1^ Canteen Australia Sydney New South Wales Australia; ^2^ Cancer Nursing Research Unit, Faculty of Medicine and Health The University of Sydney Sydney New South Wales Australia

**Keywords:** acceptance and commitment therapy, adolescents, cancer, program evaluation, psychological flexibility, psycho‐oncology, self‐compassion

## Abstract

PEER is a four‐day residential program for adolescents impacted by their own or a relative's cancer, with both psychosocial (acceptance and commitment therapy, self‐compassion) and recreational components. This study aimed to determine whether previously observed improvements in quality of life amongst highly distressed participants were mediated by improvements in processes targeted by psychotherapeutic elements of the program (psychological flexibility, mindfulness, self‐compassion, peer support, distress). Adolescents attending PEER completed surveys assessing the quality of life and proposed mediator variables at pre‐program, post‐program and two‐month follow‐up. Adolescents experiencing high/very high levels of baseline distress (*n* = 52; 5 patients/survivors, 31 siblings/offspring, 13 bereaved siblings/offspring) were previously identified as experiencing clinically significant improvements in psychosocial well‐being; here, mediation analyses explored whether these improvements were associated with improvements in process variables. Findings evidenced improvements in quality of life amongst distressed PEER participants, mediated by increases in psychological flexibility and self‐compassion, and reductions in distress. Peer support and mindfulness were not significant mediators. Together, this suggests that the psychosocial benefits of PEER observed for highly distressed adolescents are linked to the specific therapeutic approaches used in the program, rather than being non‐specific effects of peer connection or recreation. Findings from this evaluation provide further evidence for the efficacy and mechanisms of the effect of PEER for supporting distressed adolescents impacted by cancer. The study also demonstrates the viability and utility of the therapeutic approaches (acceptance and commitment therapy, self‐compassion) used, showing that they have psychosocial benefits for this population.

**What is known about this topic?**

- Acceptance and Commitment Therapy (ACT) and self‐compassion show promise in facilitating adolescents' adjustment to their own or their parent/sibling's cancer.
- PEER is an ACT‐ and self‐compassion‐based program developed for adolescents impacted by cancer.
- Highly distressed participants have been shown to experience greater improvements in quality of life after participating in PEER.

**What this paper adds**

- Highly distressed PEER participants experienced improved quality of life from baseline to two‐month follow‐up, mediated by changes in therapeutic processes (psychological flexibility, self‐compassion).
- The benefits of the PEER program are linked to the specific therapeutic approaches used, rather than peer connection or recreation.
- ACT and self‐compassion have demonstrated psychosocial benefits for distressed adolescents impacted by cancer.

## 1 INTRODUCTION

For adolescents, a personal or familial cancer diagnosis can cause significant psychosocial disruption at an already dynamic developmental life stage. Just over 40% of young people diagnosed with cancer experience clinical levels of distress (Patterson, D'Agostino, et al., 2021); anxiety and post‐traumatic stress symptoms, feelings of difference from peers, and difficulties integrating their cancer experience with their personal identity are also common (Baird et al., 2019; Kim et al., 2016). Those whose parent or sibling has, or has died from, cancer can also experience elevated levels of distress and unmet needs (Long et al., 2018; Patterson, McDonald, Ciarrochi, et al., 2017; Varathakeyan et al., 2017; Walczak et al., 2017). Common to the experiences of adolescent patients/survivors, offspring and siblings is the need for help adjusting to their cancer situation, and for support from other young people who are similarly affected (Kaluarachchi et al., 2020; McDonald et al., 2014; Walczak et al., 2017).

Residential (multi‐day/overnight, live‐in) programs have been identified as a promising format of psychosocial intervention for adolescents impacted by cancer, as they combine the chance to ‘escape’ the immediate cancer situation with opportunities for peer connection, support and learning coping strategies (Clarke et al., 2021; Hancock, 2011; Martiniuk et al., 2014; Patterson, McDonald, Kelly‐Dalgety, Lavorgna, et al., 2021; Patterson, McDonald, Kelly‐Dalgety, Luo, & Allison, 2021; Wu et al., 2011). Whilst some previous evaluation research has evidenced the potential for such programs to improve psychosocial well‐being (e.g. Antonetti et al., 2020; Packman et al., 2005; Patterson, McDonald, Kelly‐Dalgety, Luo, & Allison, 2021), few studies have examined the mechanisms underpinning their therapeutic benefits. This paper presents a mediation analysis to build on a previous process evaluation of the acceptance and commitment therapy (ACT)‐based PEER program (Patterson, McDonald, Kelly‐Dalgety, Luo, & Allison, 2021), to determine whether improvements in quality of life observed in highly distressed participants are associated with changes in ACT process variables.

### 1.1 Acceptance and commitment therapy

Like other third‐wave cognitive‐behavioural therapeutic approaches, the focus of ACT lies in how individuals *relate* to their thoughts and emotions, rather than their specific contents (Marshall & Brockman, 2016). The approach aims to improve psychological flexibility, which describes an individual's ability to allow themselves to experience their emotions, rather than attempting to challenge, change or avoid unwanted thoughts and feelings (Hayes et al., 2006; Hulbert‐Williams et al., 2015). This is developed through six key processes (the hexaflex model): acceptance of thoughts and emotions, self as context (distinguishing mental states from the self), cognitive defusion (weakening the influence of mental states on behaviour), mindfulness, defining values and committing to values‐consistent actions (Hayes et al., 2006; Hulbert‐Williams et al., 2015). These processes have been linked to improved psychosocial well‐being in adolescents (Halliburton & Cooper, 2015), including those with chronic health conditions (Ernst & Mellon, 2016) such as cancer (Patterson & McDonald, 2015), and offspring impacted by parental cancer (Patterson, McDonald, White, et al., 2017). Indeed, ACT has been identified as particularly promising for cancer‐affected populations (Clarke et al., 2020; González‐Fernández & Fernández‐Rodríguez, 2019; Ing et al., 2019), where difficult emotions and cognitions may be reasonable responses to ongoing health risks and challenges (e.g. fear of cancer recurrence) and where learning to live with these internal processes may better facilitate adjustment to the cancer situation (Hulbert‐Williams et al., 2015).

ACT‐based programs have been demonstrated to influence the underlying therapeutic processes, such as psychological flexibility and experiential avoidance (avoiding difficult thoughts, feelings and internal experiences—a key process of psychological inflexibility)(Feros et al., 2013; Halliburton & Cooper, 2015; Patterson, McDonald, White, et al., 2017), and thereby elicit improvements in psychosocial well‐being (Feros et al., 2013; Patterson, McDonald, White, et al., 2017). Of particular relevance are studies demonstrating the process of change for individuals impacted by cancer participating in ACT‐based interventions: for example, a nine‐week ACT intervention was found to produce lasting improvements in quality of life and emotional well‐being amongst cancer patients, mediated by increased psychological flexibility (Feros et al., 2013), whilst another ACT program demonstrated decreases in depression associated with reduced experiential avoidance amongst young people impacted by parental cancer (Patterson, McDonald, Ciarrochi, et al., 2017; Patterson et al., 2015). Likewise, González‐Fernández and Fernández‐Rodríguez's (2019) review of 19 studies examining the effects of ACT in the cancer context indicated psychosocial benefits of this therapeutic approach, although they note the limited ability to identify the specific mechanisms underlying these improvements as a limitation of this literature. This is particularly the case where ACT processes may not be the only variables impacted by an intervention (e.g. group‐based interventions may also improve well‐being through peer support), or where different therapeutic approaches are used in combination.

### 1.2 Mindfulness

Whilst mindfulness is conceptualised as a core component of ACT (Hayes et al., 2006; Hulbert‐Williams et al., 2015), it can also be used as an independent therapeutic approach. Its focus on deliberate, non‐judgemental presence at the moment (Kabat‐Zinn, 2003) may be useful in helping young people impacted by cancer to adapt to their situation and cope with their thoughts and emotions (Patterson & McDonald, 2015). Preliminary evidence suggests that mindfulness‐based interventions are feasible, acceptable and improve psychological well‐being amongst AYA patients/survivors (Nissim et al., 2020; Van der Gucht et al., 2017). Therefore, in evaluating the impacts of ACT‐based interventions, it may be informative to compare the contribution of mindfulness to those of other mechanisms unique to ACT (e.g. cognitive flexibility).

### 1.3 Self‐compassion

Combining ACT with other complementary approaches may also enhance therapeutic benefits for participants (Marshall & Brockman, 2016; Patterson, McDonald, Kelly‐Dalgety, Luo, & Allison, 2021). Self‐compassion may facilitate adjustment to cancer by encouraging identification and non‐judgemental response to personal struggles (mindfulness), finding commonalities in challenges (common humanity) and being understanding to oneself during difficult times (self‐kindness)(Neff, 2003a). Often used in combination with mindfulness, interventions informed by self‐compassion have been shown to be feasible and effectively improve psychosocial well‐being for healthy adolescents (Bluth et al., 2016; Bluth & Eisenlohr‐Moul, 2017; Rodgers et al., 2018) and young adults diagnosed with cancer (Campo et al., 2017; Lathren et al., 2018)—although the approaches appear to have distinct impacts on well‐being (Marshall & Brockman, 2016), and may, therefore, offer greater therapeutic benefits when used in combination.

### 1.4 PEER: A place for enablement, empowerment and relationships

PEER is a four‐day therapeutic and recreational program for adolescents (12–17 years) impacted by their own or a relative's cancer, which draws from ACT and self‐compassion in its therapeutic approach. The program involves seven psychosocial sessions (*Building Supportive Relationships; Listening to Each Other's Stories; Effective Coping; Luminescence; Be Kind to Yourself; We Are All In This Together; Reach Out*) delivered to groups of 3–10 adolescents by two facilitators trained in ACT and experienced in providing support to this population. These sessions were interspersed with group recreational activities (see Patterson, McDonald, Kelly‐Dalgety, Luo, & Allison, 2021 for further details of the development, structure and content). A preliminary evaluation of the program indicated that it was delivered with good fidelity, with high levels of participant satisfaction and engagement (Patterson, McDonald, Kelly‐Dalgety, Luo, & Allison, 2021). Further, between 15 and 20% of participants experienced clinically meaningful improvements in distress and quality of life between pre‐program and two‐month follow‐up, with moderation analyses indicating that these psychosocial benefits were greatest for participants with higher distress and lower psychological flexibility, mindfulness and self‐kindness at baseline (Patterson, McDonald, Kelly‐Dalgety, Luo, & Allison, 2021). That is, the program appears to be most effective for those in greatest need of coping strategies to help adapt to their cancer experience. However, the extent to which these improvements are linked to the specific therapeutic processes targeted by the program (as opposed to the general benefits of peer support and connection) remains unclear.

### 1.5 Present study

This paper reports on the processes of change underlying improvements in quality of life (QOL) observed amongst PEER attendees who presented with high or very high levels of distress at the beginning of the program, as this group was previously noted to derive the greatest benefit from program participation (Patterson, McDonald, Kelly‐Dalgety, Luo, & Allison, 2021). To better understand the mechanisms underlying these improvements, we use mediation analyses to evaluate whether changes in QOL are attributable to changes in the therapeutic processes targeted by PEER (psychological flexibility, self‐compassion, mindfulness), over and above changes in peer support associated with purely recreational programs. Since this was a highly distressed sample, we also examined whether improvements in quality of life are secondary to improvements in distress, or whether they occur independently of any change in distress (which may be influenced by external factors such as the progress of cancer). We did not treat distress as a primary outcome in this paper, since the selection of a highly distressed sample could artificially increase any obtained improvements in this outcome due to regression to the mean (Barnett et al., 2004).

## 2 METHODS

### 2.1 Participants

As previously reported, the 2017 evaluation of PEER involved 148 adolescent participants who attended the program and completed the evaluation at two or more time points (Patterson, McDonald, Kelly‐Dalgety, Luo, & Allison, 2021). This paper reports on 52 program participants who completed measures at all three time points and had high to very high levels of distress (Australian Bureau of Statistics, 2012)^1^ before beginning the program. These participants were aged between 12 and 17 years, predominantly female (79%), and most had a sibling/parent with cancer (63%). Further demographic details are presented in Table 1. Compared to participants experiencing low to medium distress before PEER, those included in these analyses were more likely to be female (*p* = 0.039), the person with cancer was more likely to still be in active treatment (*p* = 0.033), and on average that person had been diagnosed more recently (*p* = 0.018). There was no difference in participant age, cancer experience category (patient/survivor, sibling/offspring or bereaved), ethnic background, employment status or length of engagement with Canteen^2^ between the high/very high participants included in this analysis and those with low/medium distress.

**TABLE 1:** Participant demographics

Demographic	*n* (%)	*M* (*SD*)
Cancer experience		
Patient/survivor	5 (10.2)	
Sibling/offspring	31 (63.3)	
Bereaved sibling/offspring	13 (26.7)	
Time since diagnosis (years)		3.5 (3.5)
Person with cancer still on active treatment	31 (60.8)	
Age at start of program (years)		15.4 (1.5)
Gender		
Female	41 (78.8)	
Male	11 (21.2)	
Born outside Australia	2 (3.9)	
Aboriginal or Torres Strait Islander	3 (5.8)	
In paid employment	17 (35%)	
Length of Canteen engagement (months)		16.1 (16.0)

### 2.2 Evaluation design

PEER was evaluated across six residential programs run by Canteen in 2017. Prior to each program, participants and their parents provided written consent for their involvement in the program evaluation. The study procedure involved the completion of a pen‐and‐paper survey at three time points: at the beginning of the program (T0), the end of the program (T1) and 2 months following program completion (T2). This survey included demographic questions^3^ and measures of the primary outcome (quality of life) and hypothesised mediators (psychological flexibility, mindfulness, self‐compassion, peer support and distress). With the exception of demographics (T0 only) and distress (T0 and T2 only^4^), all other measures were completed at all three time points. The study procedure was approved by the University of Sydney Human Research Ethics Committee (2017/104).

### 2.3 Measures

#### 2.3.1 Primary outcome: Quality of life (QOL)

QOL was measured using a single item asking “How happy are you with your life as a whole?”. Participants responded using an 11‐point Likert scale (*very sad/unsatisfied* to very *happy/satisfied*), with scores ranging from 0 to 10). This item is derived from the Personal Well‐being Index‐School Children (Cummins & Lau, 2005) with which it is strongly correlated, demonstrating convergent validity (Tomyn et al., 2013); more generally, single‐item measures have been successfully used to assess QOL, including in the cancer context (Bush et al., 2010; de Boer et al., 2004; Wasson, 2019), with 11‐point scales appearing to have the highest reliability and validity (Kroh, 2006; Organisation for Economic Co‐operation and Development, 2013).

#### 2.3.2 Process variables: Psychological distress

Distress was assessed using the Kessler Psychological Distress Scale (K10; Kessler et al., 2002), which comprises 10 items assessing the frequency of distressing feelings in the past 30 days. Participants respond using a 5‐point Likert scale (*none of the time* to *all of the time*), with scores summed to produce an overall distress score (range 10–50). Following Australian Bureau of Statistics guidelines, scores are categorised as indicating low (10–15), moderate (16–21), high (22–29) or very high (30–50) distress (Australian Bureau of Statistics, 2012). The K10 is used widely in Australian adolescent mental health surveys (Hafekost et al., 2016) and service evaluations (Bassilios et al., 2017), and has shown adequate reliability and good predictive validity for depression in Australian adolescents.

#### 2.3.3 Peer support

The 11‐item Cancer Peer Support Scale (CaPSS; Patterson et al., 2018) was used to assess the extent to which participants felt they were supported by other young people with a cancer experience. Individuals respond to statements (e.g. “*I have been understood*”) using a 5‐point Likert scale (*none of the time* to *all of the time*) to rate how often they have felt supported by peers; total scores range from 11 to 55. Preliminary work with this measure has established its validity and reliability with young people attending therapeutic/recreational programs (Patterson et al., 2018).

#### 2.3.4 Psychological flexibility

The 8‐item version of the Avoidance and Fusion Questionnaire—Youth (AFQ‐Y8; Greco et al., 2008) assesses psychological inflexibility and was reversed‐scored in this study to provide a measure of psychological flexibility. Participants rate their agreement with statements about how they relate to their emotions (e.g. “*my thoughts and feelings mess up my life*”) using a 5‐point Likert scale (*not at all true* to *very true*). Total scores range from 0 to 32. The AFQ‐Y8 has been demonstrated to be more psychometrically robust and more accurate at classifying clinical depression and anxiety than the original 17‐item measure (Renshaw, 2017).

#### 2.3.5 Self‐kindness

The five‐item self‐kindness subscale of the Self‐Compassion Scale (Neff, 2003c) was used to measure this construct. Participants were asked to rate how often they engage in different behaviours (e.g. “I'm kind to myself when I'm experiencing suffering”) on a 5‐point Likert scale (*almost never* to *almost always*). Total scores ranged from 5 to 25. The full scale has demonstrated reliability, validity and theoretical coherence, including in adolescents (Cunha et al., 2016; Neff, 2016), and the self‐kindness scale specifically has adequate reliability, and good convergent and divergent validity (Cunha et al., 2016).

#### 2.3.6 Mindfulness

The 10‐item version of the Child and Adolescent Mindfulness Measure (CAMM; Greco et al., 2011) was used to assess mindfulness. Participants rated how true each item (e.g. “*I push away thoughts I don't like*”) was for them, using a 5‐point Likert scale (*never true* to *always true*). Total scores range from 0 to 40, with higher scores indicating *lower* mindfulness. The CAMM has demonstrated reliability and confirmed single‐factor structure with adolescents (Kuby et al., 2015).

#### 2.3.7 Other measures

Participants and facilitators completed measures of program fidelity (training quality, adherence and engagement) and satisfaction, as previously reported (Patterson, McDonald, Kelly‐Dalgety, Luo, & Allison, 2021), as well as the Brief COPE measure of coping strategies (Carver, 1997) and self‐judgement subscale of the self‐compassion measure (Neff, 2003a). These measures were not used in these analyses.

### 2.4 Data analysis

Data analysis involved two separate procedures. First, we examined the change in distressed participants' quality of life over time using a mixed linear model, to confirm that the PEER program was associated with significant improvements in this outcome. Then, we examined possible mechanisms underlying this improvement using mediation analyses. All analyses were conducted in IBM's Statistical Package for the Social Sciences (SPSS). As missing data was minimal (maximum 3% for each item), values were not imputed for incomplete items.

#### 2.4.1 Analysis of changes in QOL for distressed participants

A mixed linear model was used to examine whether distressed participants' QOL improved across the three time points (pre, post and two‐month follow‐up), with regression coefficients treated as random variables. Three models were tested: (1) a linear effect of time on QOL with no additional covariates or factors; (2) a quadratic effect of time on QOL with no additional covariates or factors; and (3) a quadratic effect of time on QOL, with demographics (age, gender, cancer experience) included as additional covariates or factors.^5^ For each, we compared model fit between (a) a fixed intercept and slope; (b) a random intercept and fixed slope; (c) a random slope and fixed intercept; (d) a random intercept and slope. Model fit was compared using information criteria values for the −2 restricted log‐likelihood (−2RLL), Akaike Information Criterion (AIC) and Bayesian Information Criterion (BIC).

#### 2.4.2 Mediation analysis

The mediation analyses were conducted using the MEMORE (MEdiation and MOderation for REpeated measures) macro for SPSS, which allows testing of mediation effects in repeated measures designs with two occasions of testing (Montoya & Hayes, 2017). We selected the pre (T0) and 2‐month follow‐up (T2) time points for this analysis. Changes in the outcome (QOL) and mediators (i.e., process variables: distress, peer support, psychological flexibility, mindfulness and self‐kindness) between T0 and T2 are assumed to be attributable to program participation (Duarte & Pinto‐Gouveia, 2017; Montoya & Hayes, 2017). Mediation effects were tested separately, given the limited sample size and the correlation between process variables (Preacher & Hayes, 2008).

## 3 RESULTS

### 3.1 Analysis of changes in QOL for distressed participants

Results of the mixed linear models exploring changes in QOL for distressed participants are presented in Tables 2 and 3. As can be seen in Table 2, Model 3b, which included linear and quadratic effects of time on QOL, demographic covariates, a random intercept and a fixed slope, demonstrated the best fit (as indicated by the lowest values of the ‐2RLL, AIC and BIC). The model accounted for 62.9% of the variance in QOL. As can be seen in Table 3, there was a significant linear effect of time on QOL in the final model, suggesting that for distressed participants, QOL increases following program participation and continues to improve across the two‐month follow‐up. No other effects reached significance in the model.

**TABLE 2:** Information criteria values comparing twelve mixed linear models of change in QOL for distressed participants completing the PEER program

Model	Parameters	−2RLL	AIC	BIC	Test	df	LRT	P
Time								
1a. Fixed	3	889.85	891.85	895.173				
1b. RI	4	812.257	816.257	822.903	1 vs 2	1	77.593	<0.01
1c. RS	4	879.446	883.446	890.092	2 vs 3	0	−67.189	
1d. RI + RS	5	811.353	817.353	827.322	2 vs 4	1	0.904	0.34
Time^2^								
2a. Fixed	4	889.531	891.531	894.849	2 vs 5	0	−77.274	
2b. RI	5	809.746	813.746	820.382	2 vs 6	1	2.511	0.11
2c. RS	5	878.797	882.797	889.434	2 vs 7	1	−66.540	
2d. RI + RS	6	808.469	814.469	824.424	2 vs 8	2	3.788	0.15
Time^2^ + Covariates								
3a. Fixed	8	840.213	842.213	845.465	2 vs 9	4	−27.956	
3b. RI	9	771.935	775.935	782.439	2 vs 10	1	40.322	<0.01
3c. RS	9	832.338	836.338	842.842	10 vs 11	0	−60.403	
3d. RI + RS	10	771.082	777.082	786.839	10 vs 12	1	0.853	0.36

Abbreviations: AIC, Akaike Information Criterion; BIC, Bayesian Information Criterion; df, degrees of freedom; LRT, log ratio test; p, p‐value; RI, Random Intercept; RS, Random slope; RI + RS, Random Intercept and Random Slope; −2RLL, −2 restricted log likelihood. Models tested were as follows: (1) linear effect of time on QOL with no additional covariates or factors; 2) quadratic effect of time (Time^2^) on QOL with no additional covariates or factors; (3) quadratic effect of time (Time^2^) on QOL, with demographics (age, gender, cancer experience) included as additional covariates or factors.

**TABLE 3:** Estimates of effects from the mixed linear model (3b) of change in QOL for distressed participants completing the PEER program, with participant ID used as a random effect

Variables	Coefficient	*SE*	*P*	95% CI
Fixed effects				
Intercept	4.39	2.05	0.036	0.30, 8.48
Time	1.09	0.37	0.004	0.35, 1.81
Time^2^	−0.35	0.19	0.061	−0.73, 0.02
Age	0.06	0.13	0.672	−0.21, 0.32
Gender				
Female	−0.92	0.047	0.055	−1.85, 0.02
Male	^a^			
Cancer experience				
Patient	1.48	0.76	0.055	−0.03, 2.99
Relative	0.49	0.45	0.275	−0.39, 1.38
Bereaved	^a^			
Random effects				
Residual	1.52	0.20	<0.001	1.18, 1.96
Intercept	2.58	0.53	<0.001	1.72, 3.87

*Note*: SE, standard error; *p*, *p*‐value; 95% CI, 95% confidence interval. Model 3b involved a quadratic effect of time (Time^2^) on QOL, with demographics (age, gender, cancer experience) included as additional covariates or factors, and a random intercept.

^a^ Reference group.

#### 3.1.1 Mediation analysis

**FIGURE 1:**
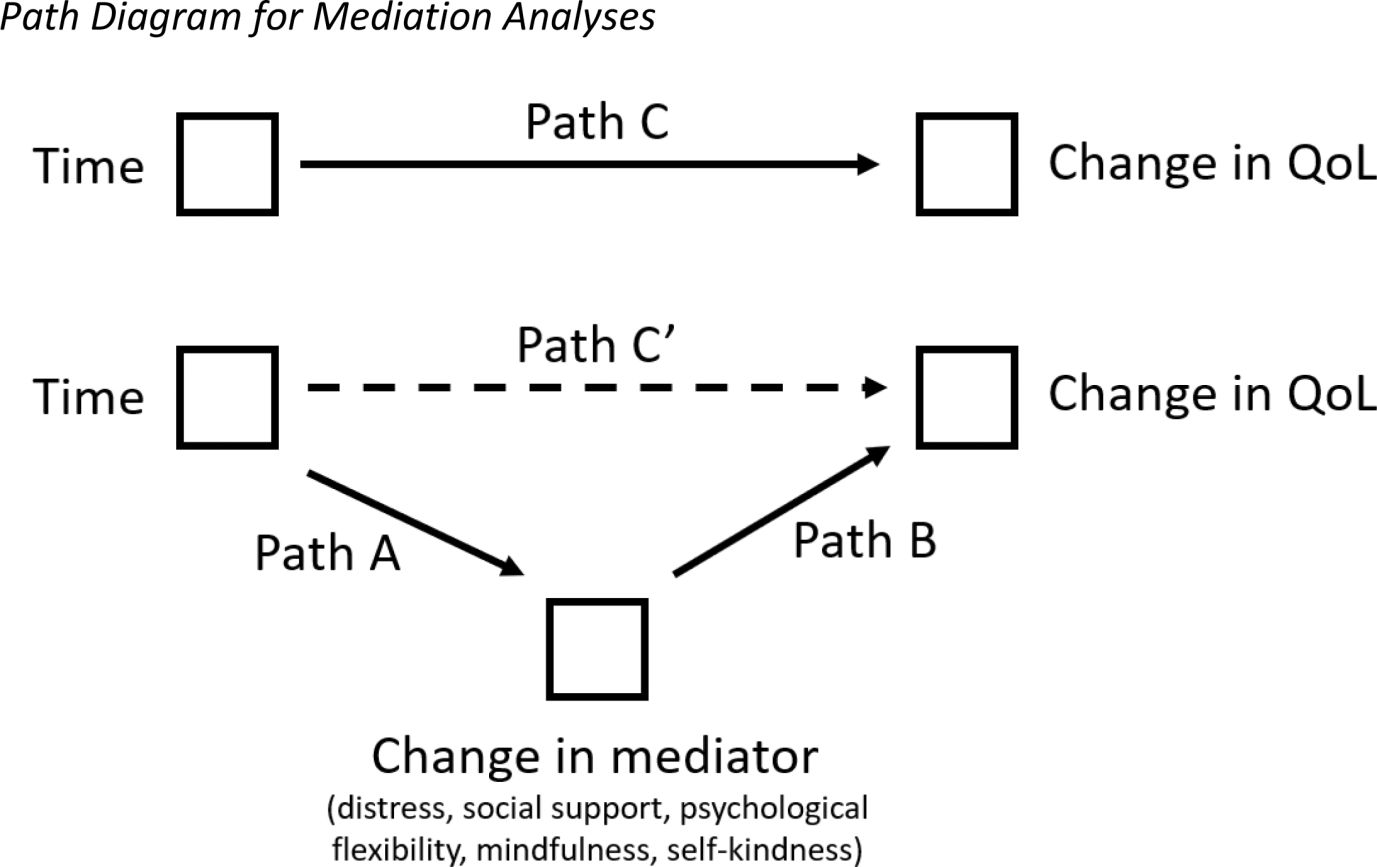
Path diagram for mediation analyses.

Figure 1 presents the path diagram of the mediation analysed, for each of the five tested mediators; Table 4 presents the standardised βs, standard errors and p‐values for each path. These indicate significant indirect effects of program participation on QOL for distressed participants, mediated by distress (*β* = 0.77, *SE* = 0.20, 95% CI [0.39, 1.19]), psychological flexibility (*β* = 0.21, *SE* = 0.11, 95% CI [0.002, 0.442]) and self‐kindness (*β* = 0.26, *SE* = 0.12, 95% CI [0.04, 0.51]). Participating in PEER is associated with reduced distress and increased psychological flexibility and self‐kindness (significant path As); changes in these mediating variables are linked to improved QOL (significant path Bs). The overall change in QOL over time (path C) was significantly altered after accounting for the indirect effect of each mediator (path C′), indicating significant mediation effects for these variables. The results show that peer support and mindfulness did not significantly mediate the effects of program participation on QOL. Together, these results suggest that the observed improvement in distressed participants' QOL after participating in PEER was specifically linked to improvements in distress, psychological flexibility and self‐kindness.

**TABLE 4:** Mediation of changes in QOL by process variables in distressed participants

Mediators	Path A β (*SE*)	Path B β (*SE*)	Path C′ β (*SE*)	Path C β (*SE*)
Distress	−4.40 (0.96)***	−0.17 (0.03)***	−0.11 (0.27)	0.66 (0.27)*
Peer support	−0.70 (0.84)	0.04 (0.05)*	0.69 (0.27)*	0.66 (0.27)*
Psychological flexibility	1.71 (0.78)*	0.12 (0.05)*	0.44 (0.27)	0.65 (0.27)*
Mindfulness	1.16 (1.01)	0.06 (0.04)	0.59 (0.27)*	0.65 (0.27)*
Self‐kindness	1.00 (0.45)*	0.26 (0.08)*	0.40 (0.26)	0.66 (0.27)*

* *p* ≤ 0.05

*** *p* ≤ 0.001.

## 4 DISCUSSION

This study explores the mechanisms underlying benefits of PEER for distressed participants, with results indicating that increases in the therapeutic processes underpinning the program—particularly psychological flexibility and self‐kindness—are linked to improvements in QOL following participation. Reduction in distress also mediated this change, whilst mindfulness and peer support were not statistically significant mediators. This builds on findings from the previous process evaluation of PEER which identified that the program resulted in clinically significant improvements in QOL and distress for some participants, with those who reported high and very high levels of baseline distress appearing to benefit more from the program (as did those with lower levels of process variables: psychological flexibility, mindfulness and self‐kindness)(Patterson, McDonald, Kelly‐Dalgety, Luo, & Allison, 2021). Together, these findings suggest that participation in the PEER program has psychosocial benefits for young people in greatest need of support in coping with their cancer experience (regardless of what this experience is), and that for this group, these benefits are attributable to the specific therapeutic teachings of the program (ACT and self‐compassion) rather than non‐specific effects of peer connection or recreation.

The evidenced mechanisms of the effect of the PEER program provide further evidence to support the use of ACT and self‐compassion as therapeutic approaches when working with adolescents impacted by their own or a family member's cancer, particularly those experiencing higher levels of distress. Whilst previous literature evidences the benefits of these approaches for young people (Bluth et al., 2016; Bluth & Eisenlohr‐Moul, 2017; Ernst & Mellon, 2016; Halliburton & Cooper, 2015; Rodgers et al., 2018; Swain et al., 2015), people with cancer (Feros et al., 2013; Hulbert‐Williams et al., 2015) and those at the intersection of these groups (Campo et al., 2017; Patterson, McDonald, Ciarrochi, et al., 2017; Patterson et al., 2019; Patterson, McDonald, Kelly‐Dalgety, Luo, & Allison, 2021) has been relatively limited, these approaches were considered promising for this population because of their focus on non‐judgemental and compassionate acceptance of difficult thoughts and feelings brought on by cancer (Clarke et al., 2020; González‐Fernández & Fernández‐Rodríguez, 2019; Hayes et al., 2006; Hulbert‐Williams et al., 2015; Ing et al., 2019). Struggling with difficult thoughts and feelings is a reasonable response to the challenging life circumstance of a cancer diagnosis that is beyond one's control; being able to accept and live well with these emotions and cognitions, rather than engaging in the futility of trying to challenge or change them, may be more adaptive. Here, improvements in QOL were tied to increases in psychological flexibility and self‐kindness after participating in PEER, suggesting that distressed adolescents who learned to accommodate their inner experiences and respond with acceptance and understanding experienced improved psychosocial well‐being as a result. This adds to previous literature linking the impact of ACT interventions to improvements in psychological flexibility (Feros et al., 2013).

Interestingly, neither mindfulness nor peer support was significant mediators of the improvement in QOL in this study. That mindfulness did not mediate this change suggests that the observed impacts of ACT for adolescents impacted by cancer are not solely attributable to this therapeutic component; that is, the six elements of ACT in combination appear to offer greater psychosocial benefit than mindfulness in isolation. Likewise, that peer support did not mediate improvements in QOL suggests that program benefits for distressed participants are not purely due to opportunities for peer support and connection. However, qualitative testimonials from participants evidenced the clear value adolescents found in connecting, sharing experiences and exchanging support with peers (reported in the previous PEER evaluation paper (Patterson, McDonald, Kelly‐Dalgety, Luo, & Allison, 2021)), and doing so may have enhanced the impact of therapeutic components of the program (Clarke et al., 2020) or benefitted participants in ways not easily captured by quantitative measures and analyses. This highlights the importance of mixed‐method approaches to program evaluation in order to more comprehensively capture experiences and impacts of psychosocial interventions, including how this may vary for different subgroups of participants.

Finally, improvements in QOL were also mediated by reductions in distress—perhaps unsurprisingly, as these participants were experiencing high to very high levels of distress at the beginning of the program. The effect sizes for this mediation were the largest of all mediators, although caution is required when interpreting this comparison, as the relatively small sample precluded entering multiple mediators in a single analysis.

### 4.1 Limitations

It is important to note that the results of this study are only true of a subset of participants: those adolescents with clinically elevated levels of distress at baseline (Australian Bureau of Statistics, 2012), who were previously identified to experience greater psychosocial benefits of program participation (Patterson, McDonald, Kelly‐Dalgety, Luo, & Allison, 2021). As discussed above and evidenced in the previous process evaluation (Patterson, McDonald, Kelly‐Dalgety, Luo, & Allison, 2021), less distressed participants may benefit in different ways or from different aspects of the program, such as reduced isolation, feelings of commonality and opportunities to share experiences with others who “get it” which are often associated with peer connection. Future work may also be strengthened through the use of a validated questionnaire (e.g. the Kidscreen‐10 [Erhart et al., 2009]) rather than a single item to assess QOL—although this may increase the survey burden for participants, particularly if multiple processes and outcome variables are being assessed.

### 4.2 CONCLUSIONS

The findings of this study not only confirm the benefits and underlying therapeutic processes of the PEER program; they also demonstrate the viability and impact of ACT and self‐compassion for adolescents who are experiencing high levels of distress as a result of their own or a family member's cancer. Improvements in QOL amongst distressed adolescents were specifically mediated by increases in psychological flexibility and self‐kindness, suggesting that therapeutic interventions can improve these processes in this population, with resulting psychosocial benefits. The finding that mindfulness and peer support did not mediate improvements in quality of life suggests that incorporating ACT and self‐compassion as part of therapeutic intervention may be more effective than programs based solely on peer support and/or mindfulness. Whilst further research comparing different interventions would be needed to confirm this, healthcare providers and community organisations may want to prioritise programs and services that incorporate ACT and self‐compassion when considering the most appropriate referrals to make, or support services to offer, distressed young people impacted by cancer.

## FUNDING INFORMATION

Canteen is a registered charity supported by government funding and donations from individuals, corporations, trusts and foundations. The evaluation of the PEER program received no specific funding.

## ACKNOWLEDGEMENTS

The authors would like to acknowledge Dr Richard Tindle for his contribution to the statistical analysis, the Canteen staff who conducted the PEER program, and all the young people who participated in the evaluation. Open access publishing facilitated by The University of Sydney, as part of the Wiley ‐ The University of Sydney agreement via the Council of Australian University Librarians.

## CONFLICT OF INTEREST

All authors on this paper are affiliated with Canteen, which owns the intellectual property rights to the PEER program; there are no other conflicting interests associated with this project.

## Data Availability

Research data are not shared due to privacy and ethical restrictions.

## Endnotes

^1^
The moderation analysis in the previous process evaluation indicated that participants with a score of 23.9 or higher on the Kessler‐10 measure of psychological distress experienced significant improvements in QOL after program participation. Here a cut‐off of 22 was used; this is more clinically meaningful, as it corresponds to the cut‐off for high or very high levels of distress (Australian Bureau of Statistics, 2012).

^2^
Canteen is an Australian not‐for‐profit organisation which supports AYAs impacted by their own or a family member's cancer (including informational, psychosocial, recreational and peer support options). The organisation developed and runs the PEER program.

^3^
Demographic information collected in the T0 survey was supplemented by additional data from Canteen client records, with participant assent.

^4^
The Kessler 10 measure of psychological distress was not included in the post‐program survey as it assesses distress over a 30‐day period, and is, therefore, inappropriate for detecting changes in distress over a four‐day program.

^5^
Process variables were not included as covariates as the smaller sample limited statistical power.
